# The Cavβ4 subunit of Cav1.2 channels antagonizes isoproterenol‐induced hypertrophy in rat cardiac muscle cells by down‐regulating miR‐183‐5p

**DOI:** 10.14814/phy2.71037

**Published:** 2026-07-28

**Authors:** Elba D. Carrillo, Erick Galicia, María C. García, Jorge A. Sánchez

**Affiliations:** ^1^ Department of Pharmacology Center for Research and Advanced Studies of the National Polytechnic Institute Mexico City Mexico

**Keywords:** Cavβ4 subunit, forkhead box O1, hypertrophy, microRNA‐183, nuclear receptor 4A2

## Abstract

The Cavβ4 subunit of voltage‐gated Cav1.2 channels regulates gene expression in neurons and cardiac cells. It increases the expression of interferon‐β–related genes in H9c2 cardiomyocytes derived from rat ventricular tissue, but the possibility that it also regulates the expression of microRNAs (miRs) remains unexplored. Furthermore, its role in cardiac hypertrophy is unknown. Although the mechanisms underlying cardiac hypertrophy have been studied extensively, the antihypertrophic response is poorly understood. We conducted quantitative reverse‐transcriptase polymerase chain reaction, western blot, and immunofluorescence experiments with H9c2 cardiomyocytes to examine the effects of Cavβ4 overexpression on isoproterenol‐induced hypertrophy; the protein abundance of the transcription factors nuclear receptor 4A2 (NR4A2) and forkhead box O1 (FOXO1), which counter agonist‐induced hypertrophic growth; and the expression of miR‐183‐5p, which targets NR4A2 and FOXO1 mRNAs. We found that the Cavβ4 subunit prevented the development of H9c2 cardiomyocyte hypertrophy, down‐regulating miR‐183‐5p expression and increasing the protein abundance of NR4A2 and FOXO1. We also observed a transient decrease in Cavβ4 mRNA expression in rat ventricles at 6 h after isoproterenol injection. These results suggest that the Cavβ4 subunit plays a channel‐independent role in the antihypertrophic response in cardiac muscle cells.

## 1 INTRODUCTION

Cardiac hypertrophy is a pathological phenomenon that develops as a result of pressure overload, prolonged exposure to catecholamines, and other pathological stimuli that increase the influx of Ca^2+^, essential for the activation of hypertrophic signaling (Mukherjee & Spinale, 1998). Although initially compensatory, it usually leads to cardiac failure and death (Morisco et al., 2001; Yu et al., 2020). Several signaling pathways, such as the calcineurin–nuclear factor of activated T cells (NFAT) pathway, are involved in the development of hypertrophy (Han et al., 2022; Nishida et al., 2024). Although the pathways that promote hypertrophic responses are well characterized, the mechanisms that antagonize these pathways have not been defined as clearly. Among these mechanisms is the suppression of pro‐hypertrophic calcineurin/NFAT signaling by forkhead box O (FOXO) transcription factors (Ronnebaum & Patterson, 2010). FOXO1 attenuates calcineurin activity in cultured cardiomyocytes (Ni et al., 2006). Nuclear receptor 4A2 (NR4A2) also opposes hypertrophy by inhibiting the activation of extracellular signal–regulated kinase (ERK) signaling and cell growth in response to β‐adrenergic stimulation, as observed in adult rat cardiomyocytes (Ashraf et al., 2019). NR4A2 exhibits a robust and transient (hours) expression increase in heart tissue in response to a single intraperitoneal injection of isoprenaline in mice (Myers et al., 2009). Adrenergic cardiac hypertrophy is mediated primarily by β‐adrenergic receptors in the heart. In vivo, isoproterenol (ISO) stimulation affects multiple cardiac signaling cascades, including the phosphoinositide 3‐kinase signaling pathway and ERK1/2, and promotes FOXO1 inactivation by phosphorylation (Morisco et al., 2001; Zhang et al., 2011). FOXO1 and NR4A2 are also regulated by microRNAs (miRs) (Liu et al., 2018; McLoughlin et al., 2014; Urbánek & Klotz, 2017; Yang et al., 2012), which are small noncoding RNAs involved in the posttranscriptional regulation of gene expression. This regulation occurs mainly via base pairing with the 3′ untranslated regions (UTRs) of target mRNAs, which usually represses protein translation (Saliminejad et al., 2019). miR‐183‐5p down‐regulates FOXO1 in H9c2 cardiomyocytes (Geng et al., 2023) and an inhibitor of miR‐183 upregulates NR4A2 in HEK cells, suggesting that NR4A2 is also a target of this miR (Gong et al., 2021).

Cardiac type‐1.2 voltage‐gated calcium (Cav1.2) channels play a fundamental role in excitation–contraction coupling (Bers, 2002) and are implicated in the development of hypertrophy (Makarewich et al., 2012). The Cav1.2 channel has a principal α1c subunit containing the pore and several auxiliary subunits, among which intracellular Cavβ plays key roles in α1c subunit trafficking and gating regulation (Dolphin, 2016). Four distinct Cavβ subunits have been detected in excitable cells, and a novel role for the Cavβ4 subunit as a nuclear protein regulating gene expression in neurons and cardiac muscle cells has been described (Carrillo et al., 2024; Tadmouri et al., 2012; Tammineni et al., 2018). In a previous study, we demonstrated that Cavβ4 increases the expression of key antiviral genes in cardiac cells (Tammineni et al., 2018). As miR‐183 is dysregulated in various viral infection contexts (Oussaief et al., 2015; Stark et al., 2012), we speculated that Cavβ4 and miR‐183 activity were related. In this study, we used H9c2 cardiomyocytes to test the hypothesis that the Cavβ4 subunit of Cav1.2 channels mitigates ISO‐induced hypertrophy. We further hypothesized that this protective effect is associated with a decrease in the expression of miR‐183‐5p and the upregulation of FOXO1 and NR4A2.

## 2 METHODS

### 2.1 Animals

All animal experiments were conducted following the guidelines of the Animal Care and Use Committee at the Center for Research and Advanced Studies of the National Polytechnic Institute (CINVESTAV, Mexico City, Mexico). A total of 52 male adult (aged 7–8 weeks) Wistar rats (RRID:RGD_13508588) weighing 230–250 g were used in this study. The rats were obtained from the Animal Production and Experimentation Unit of CINVESTAV. They were housed in groups of 3–4 per cage in a room maintained at 21–24°C with a 12/12‐h light/dark cycle and fed standard rat chow (5053‐PicoLab Rodent Diet 20, LabDiet, Saint Louis, MO, USA). Rats were euthanized by intraperitoneal injection of sodium pentobarbital. Only healthy animals were selected for experimentation. All experiments were performed in accordance with ARRIVE guidelines.

### 2.2 Hypertrophy models

We used isoproterenol to generate cardiac hypertrophy in adult rats and in H9c2 cardiomyocytes maintained in culture as described below.

### 2.3 Cardiac hypertrophy model in rats

Cardiac hypertrophy was induced by daily subcutaneous injections of ISO (5 mg/kg body weight, 1747; Tocris Bioscience, Bristol, UK) or vehicle (0.9% NaCl saline solution), as described elsewhere (Velusamy et al., 2020). Animals were assigned to experimental groups using simple randomization. After predetermined times, rats were anesthetized by intraperitoneal injection of 50 mg/kg pentobarbital sodium, and their hearts were rapidly excised. Ventricle fragments were immediately submerged in RNAlater (76,106, Qiagen, Germantown, MD, USA) and stored at −70°C for evaluation by quantitative reverse‐transcriptase polymerase chain reaction (qRT‐PCR).

### 2.4 Cell culture, Cavβ4 overexpression, and ISO treatment

Cell culture and transfection were performed as described elsewhere (Tammineni et al., 2018). In brief, H9c2 cells (RRID:CVCL_0286) (passages 17–24; American Type Culture Collection, Manassas, VA, USA) were cultured in monolayers in Dulbecco's modified Eagle's medium (DMEM; Gibco, Thermo Fisher, Waltham, MA, USA) supplemented with 10% fetal bovine serum (FBS; Gibco, Thermo Fisher, Waltham, MA, USA), sodium bicarbonate (1.5 g/L), penicillin (50 IU), and streptomycin (50 μg/mL; Thermo Fisher, Waltham, MA, USA) under atmospheric conditions with 5% CO_2_ at 37°C in a humidified incubator. Cells were seeded on 13‐mm coverslips (density, 40,000/coverslip) and cultured for 24 h in DMEM containing 10% FBS, then transiently transfected with pSG5 plasmid empty vector or with the same vector containing a plasmid encoding the Cavβ4 subunit (pSGB4) cloned by Castellano et al. (1993). Transfections were done with Lipofectamine 2000 (11,668,019; Invitrogen, Carlsbad, CA, USA), according to the manufacturer's instructions. The transfection efficiency was 80–90%. Transfected cells were maintained for 6 h in DMEM, containing 10% FBS, and the medium was then replaced with DMEM containing 1% FBS. Cells were maintained in this low serum medium for 18 h. Thereafter, 50 μM ISO (1747; Tocris Bioscience, Minneapolis, MN, USA) was added to induce H9c2 cardiomyocyte hypertrophy. The medium was replaced after 24 h with fresh DMEM containing 1% FBS and ISO, and the cultures were maintained for another 24 h.

### 2.5 Measurement of cell area

H9c2 cardiomyocytes seeded on coverslips were washed three times with phosphate‐buffered saline (PBS), then fixed with 95% ethanol for 20 min. Next, they were washed three times with PBS, stained with hematoxylin solution (H3136; Sigma‐Aldrich, Burlington, MA, USA) for 2 min, and washed again. Eosin solution (318,906; Sigma‐Aldrich) was then added for 1 min, and the cells were washed with water. Coverslips were mounted using 90% glycerol in PBS at pH 9 with 0.05% sodium azide. Cells were photographed under a microscope (DMI 6000 CS; Leica, Wetzlar, Germany with a dry N Plan 40x objective, NA 0.65) using a color digital camera (DFC450; Leica), and cell areas were measured using ImageJ (RRID:SCR_003070) software [ver. 2.7.0; National Institutes of Health (NIH), Bethesda, MD, USA] (Baviskar, 2011). A total of three independent experiments were conducted. In each experiment, cells were transfected under four experimental conditions: pSG5 (control), pSG5 + ISO, Cavβ4, Cavβ4 + ISO. Measurements of cell areas were done in 6–9 fields in each condition. All analyses were performed blinded such that experimenters performing data analysis were unaware of the treatments.

### 2.6 miR assays

Total RNA was isolated from H9c2 cardiomyocytes using an miRNeasy mini kit (217,004; Qiagen, Hilden, Germany) that uses QIAzol as lysis reagent and quantified by spectrophotometry (NanoPhotometer N60/N50; Implen, Munich, Germany). Standard TaqMan™ miR assays (cat. no. 4427975; Applied Biosystems, Foster City, CA, USA), which employ target‐specific stem‐loop reverse transcription primers for 3′ extended templates, were used to determine the relative expression levels of the 5p strand of miR‐183 (hsa‐miR‐183‐5p; assay ID 002269) with TaqMan Universal PCR master mix, no AmpErase UNG (cat. no. 4324018) and an iCycler iQ device (Bio‐Rad, Hercules, CA, USA). miR expression was assessed relative to the small nucleolar RNA U87 (cat. no. 001712; Applied Biosystems), as recommended by the manufacturer. Changes in expression level were determined using the 2^−ΔΔCT^ method (Livak & Schmittgen, 2001).

### 2.7 qRT‐PCR assays

Total RNA was isolated from H9c2 cardiomyocytes that had been plated and transfected as described above. Complementary DNA was synthesized using a procedure described elsewhere (Carrillo et al., 2024). To quantify mRNA, we used TaqMan assays (Applied Biosystems), an iCycler iQ device (Bio‐Rad), TaqMan gene expression master mix (cat. no. 4369016), and the primer‐probe sets for myosin heavy chain 7b (MYH7B) mRNA (Myh7b; cat. no. 4331182, ID Rn01536269_m1), FOXO1 mRNA (Foxo1; cat. no. 4331182, ID Rn01494868_m1), Cavβ4 mRNA (Cacnb4; cat. no. 4331182, ID Rn01449787_m1), and atrial natriuretic peptide (ANP) mRNA (Nppa; cat. no. 4331182, ID Rn00664637_g1). Myh7b expression was examined because its downregulation is associated with hypertrophy (Warkman et al., 2012), and Nppa was examined because it is a cardiac stress marker. Eukaryotic 18S ribosomal RNA (ID Hs99999901_s1) was used as an internal control. Quantification was performed using the 2^−ΔΔCT^ method (Livak & Schmittgen, 2001). To obtain total RNA from ventricular tissue, frozen fragments were macerated in liquid nitrogen and pulverized with a mortar. Thereafter, complementary DNA was synthesized and the mRNA expression of ANP and Cavβ4 was quantitated following the same procedure as that described above for H9c2 cardiomyocytes.

### 2.8 Western blotting

H9c2 cardiomyocytes were transfected in p35 or p60 plates and then scraped and placed in lysis buffer [150 mM NaCl, 50 mM Tris–HCl (pH 7.4), and 0.5% NP‐40] supplemented with protease and phosphatase inhibitor cocktails (78,438; Halt™; Thermo Scientific, Waltham, MA, USA). The lysates were kept on ice for 1 h and vortexed every 10 min. The samples were then centrifuged at 16,000 × *g* and 4°C for 15 min, and the supernatants were stored in liquid nitrogen until further use. Isolated protein contents were quantified using Bradford's method (Bradford, 1976). For the preparation of the protein reagent Coomassie Brilliant Blue G‐250 (1442C; Research Organics Inc., Cleveland, OH, USA), ethanol (459,844; Sigma‐Aldrich) and phosphoric acid (695,017; Sigma‐Aldrich) were used following the procedures described by Bradford (1976). Equal amounts of proteins (30–40 μg) were resolved by sodium dodecyl sulfate polyacrylamide (SDS‐Page) gel electrophoresis (pore size 0.45 μm). Sample preparation for SDS‐PAGE analysis consisted of denaturing the sample with heat (95°C for 3 min) in the presence of 2% SDS and the reducing agent β‐mercaptoethanol (1%). Proteins were transferred to a nitrocellulose membrane (Bio‐Rad) and immunoblotted with appropriate antibodies. The membranes were blocked with 4.5% nonfat milk in Tris‐buffered saline (TBS) and incubated overnight at 4°C with primary antibody. Then, they were washed in TBS containing 0.1% Tween 20 and incubated in horseradish peroxidase (HRP)‐conjugated secondary antibody for 1 h at room temperature. Chemiluminescence was detected with Immobilon western chemiluminescence HRP substrate (WBKL50500; Millipore Co., Billerica, MA, USA) and film plates (8,225,526; Carestream, Rochester, NY, USA). The areas and densities of bands in western blots were measured with ImageJ software, and the integrated density was calculated as the product of the area and mean density. The Cavβ4, NR4A2 and FOXO1 integrated density values were divided by the corresponding integrated densities of GAPDH or actin bands to obtain the protein abundance. The list of antibodies used in the present study is given in Table S1.

### 2.9 Immunofluorescence

H9c2 cardiomyocytes were grown on coverslips in 24‐well plates for confocal microscopic analysis. They were fixed with 4% paraformaldehyde (158,127; Sigma‐Aldrich) at room temperature (20–22°C), then washed for 5 min with PBS containing 1% BSA (Sigma‐Aldrich) and washed again a total of five times. Cells were then permeabilized for 10 min with 1% Triton X‐100 (X100; Sigma‐Aldrich) and washed again five times with PBS and then blocked with 1% BSA and 91% Triton X‐100 in PBS for 30 min at 4°C. The list of antibodies used in the present study is given in Table S1. Primary antibody was applied in PBS with 1% BSA and secondary antibody was applied in PBS. Permeabilized cells were incubated overnight at 4°C with primary antibody, and the experimental procedures were then conducted at room temperature. Cells were incubated with the secondary antibody for 1 h and the nuclei were counterstained with Hoechst 33342 dye (1:1000, H3570; Thermo Fisher, Waltham, MA, USA). Anti‐fade fluorescence mounting medium (Vectashield H1000, 3 μL; Vector Laboratories, Newark, CA, USA) was added to the center of each microscope slide. Confocal scanning microscopy was performed with argon (488‐nm) and helium/neon (543‐nm) lasers (TCS‐SP8; Leica with a 40x Plan Neofluar 40x objective, NA 1.25) and an optimized pinhole diameter. Confocal images were created with the LAS AF 2.6.0 software (build 7268; Leica) and analyzed using ImageJ (RRID:SCR_003070) software (ver. 2.7.0; NIH). To measure nucleus and cytoplasm fluorescence, we used image segmentation and mask‐based calculations with ImageJ following a general procedure described elsewhere (Kelley & Paschal, 2019), with minor modifications described by Carrillo et al. (2024). A total of three independent NRF4A2 experiments were conducted. In each experiment cells were transfected under two experimental conditions, pSG5 (control) and overexpressed Cavβ4. Measurements of NR4A2 fluorescence were done in 5–9 fields under control conditions and 4–9 in Cavβ4 transfected cells. FOXO1 fluorescence was measured in four independent experiments in cells transfected under control (pSG5) and overexpressed Cavβ4 conditions. A total of 4–11 fields was analyzed in each condition. All analyses were performed blinded such that experimenters performing data analysis were unaware of the treatments.

### 2.10 Statistical analysis

The results are expressed as means ± standard errors of the mean. Independent *t*‐tests were used to compare data between groups, and analyses of variance followed by Dunnett's multiple‐comparison test was used to compare more than two treatments with a control. The analyses were performed using GraphPad Prism (RRID:SCR_002798) 4.0 (GraphPad Software, San Diego, CA, USA) and a significance criterion of *p* < 0.05.

## 3 RESULTS

### 3.1 The Cavβ4 subunit antagonizes hypertrophy

**FIGURE 1:**
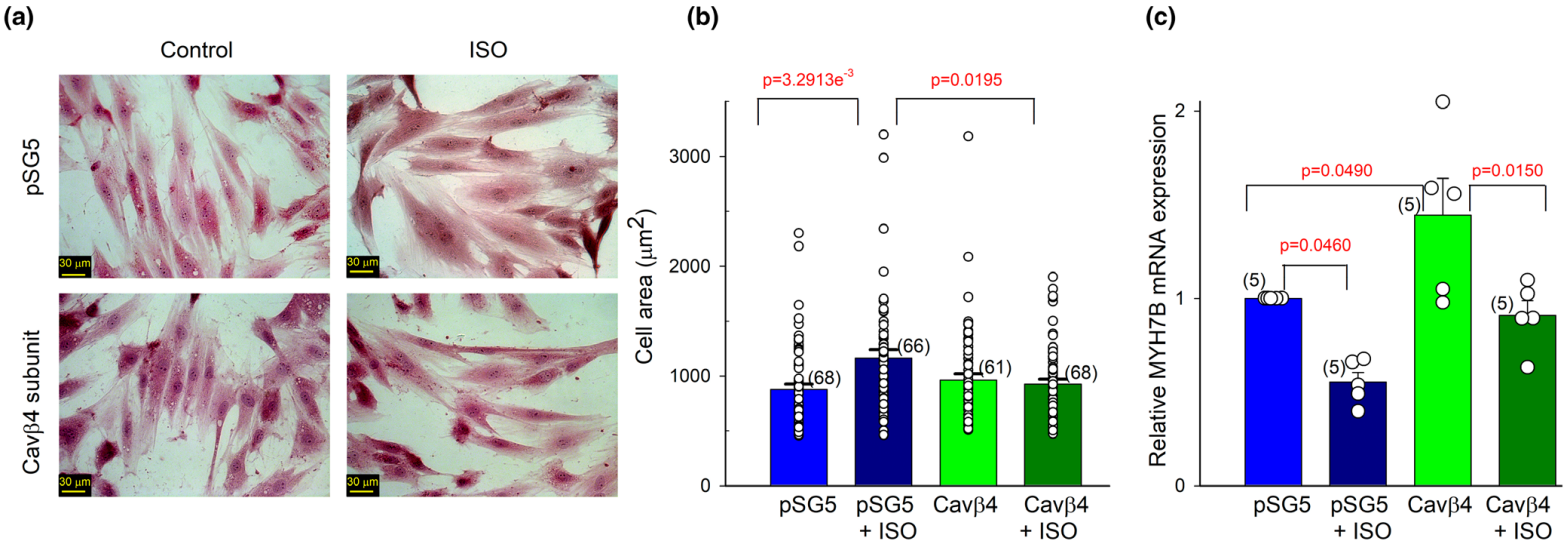
The Cavβ4 subunit antagonizes ISO‐induced hypertrophy. (a) Representative images and (b) mean (± standard error of the mean) sizes of H9c2 cardiomyocytes under control (875.9484 ± 48.0601), ISO treatment (1161.4836 ± 76.8476), Cavβ4 overexpression (961.3513 ± 57.3100), and Cavβ4 overexpression plus ISO (923.9551 ± 47.5779) conditions. The numbers of cells are indicated in parentheses in (b). (c) Relative MYH7B mRNA expression under control (1.0004 ± 1.3315e^−4^), ISO treatment (0.5906 ± 0.0456), Cavβ4 overexpression (1.5427 ± 0.2192), and Cavβ4 overexpression plus ISO (0.9117 ± 0.1025) conditions. Each symbol represents a separate experiment. The numbers of experiments are indicated in parentheses.

ISO increased the size of H9c2 cardiomyocytes transfected with the pSG5 empty vector, but it did not change that of cells transfected with the Cavβ4 subunit (Figure 1a,b), suggesting that the subunit had an antihypertrophic effect. In support of this observation, ISO reduced the expression of MYH7B in pSG5‐transfected cells, and this expression was significantly increased in cells overexpressing the Cavβ4 subunit. The addition of ISO decreased MYH7B expression in these cells to a level similar to that in pSG5‐transfected cells in the absence of ISO (Figure 1c).

### 3.2 The Cavβ4 subunit down‐regulates miR‐183‐5p and upregulates NR4A2 expression

**FIGURE 2:**
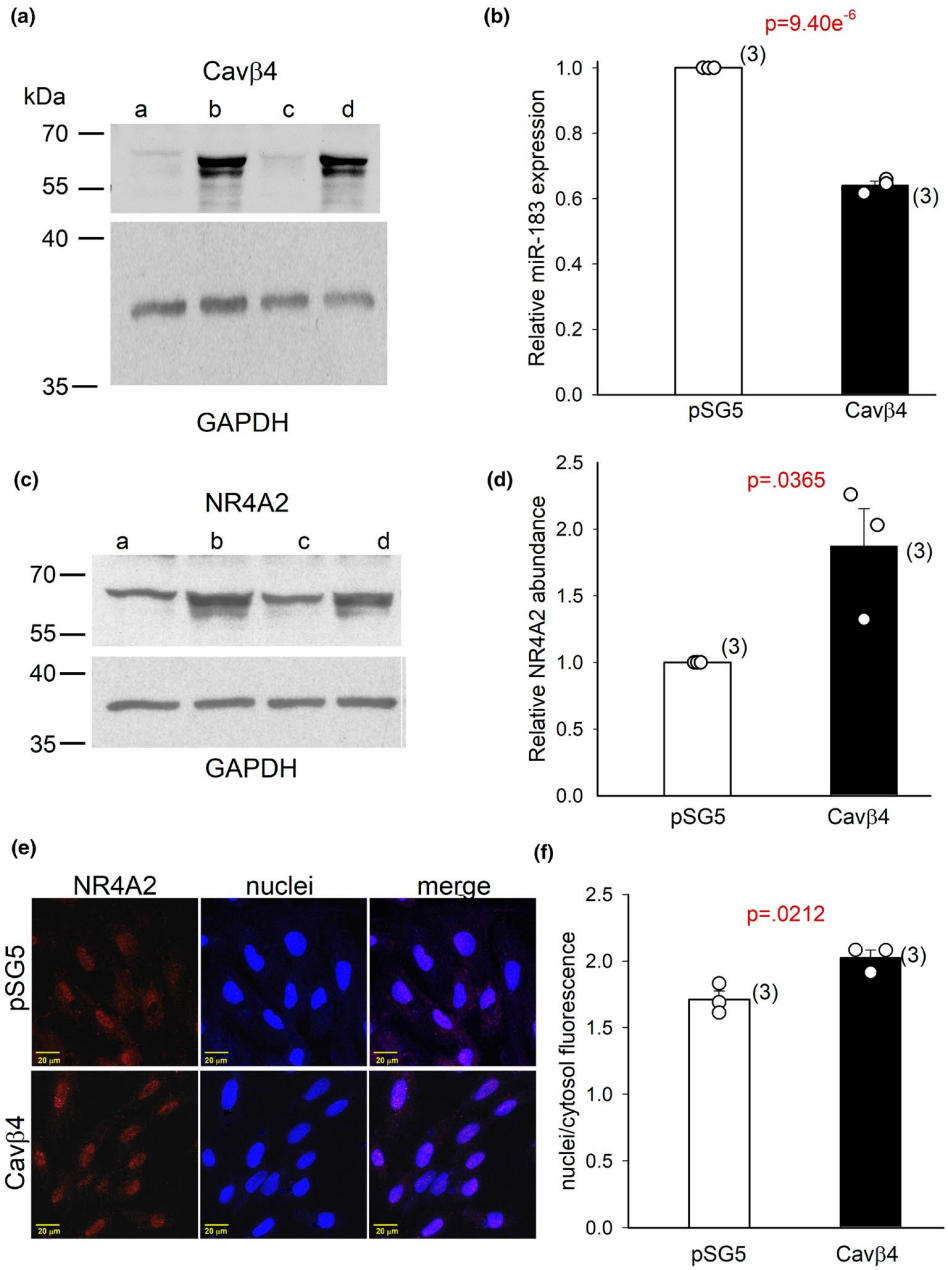
The Cavβ4 subunit decreases miR‐183‐5p and upregulates NR4A2 expression. (a) Representative blots of the Cavβ4 subunit and GAPDH under control conditions (lanes a and c) and with Cavβ4 overexpression (lanes b and d) from two separate experiments. (b) Mean (± standard error of the mean) relative miR‐183‐5p expression under control (1.004 ± 1.76e^−4^) and Cavβ4 overexpression conditions (0.6408 ± 0.0127). (c) Representative blots of NR4A2 and GAPDH under control conditions (lanes a and c) and with Cavβ4 overexpression (lanes b and d) from two separate experiments. (d) Mean (± standard error of the mean) relative NR4A2 protein abundance under control (1.000 ± 0.0414) and Cavβ4 overexpression conditions (1.8716 ± 0.2820). (e) Representative images of the immunofluorescence of H9c2 cardiomyocytes transfected with empty vector or the Cavβ4 subunit. Images show the localization of NR4A2 (red) and Hoechst‐stained nuclei (blue). (f) Mean (± standard error of the mean) NR4A2 fluorescence (nucleus/cytosol ratios) under control (1.7123 ± 0.0645) and Cavβ4 overexpression conditions (2.0268 ± 0.0560). Each symbol represents a separate experiment. The numbers of experiments are indicated in parentheses. Calibration bar, 20 μm.

The possibility that the antihypertrophic effect of the Cavβ4 subunit involves changes in miR‐183‐5p expression was investigated next in Cavβ4 overexpression experiments. In Cavβ4‐expressing cells, the antibody used recognized two protein bands, as previously described (Tammineni et al., 2018). The presence of a second band is possibly associated with posttranslational modifications (Tammineni et al., 2018). As expected, Cavβ4 density of both bands in western blots increased markedly after Cavβ4 transfection, whereas the density of GAPDH bands remained unchanged (Figure 2a). miR‐183‐5p expression decreased significantly with Cavβ4 overexpression (Figure 2b). Cavβ4 increased NR4A2 protein abundance at 48 h after transfection (Figure 2c,d), consistent with the previous demonstration that NR4A2 is a target of miR‐183 (Gong et al., 2021). GAPDH bands remained unchanged with Cavβ4 overexpression and were used for normalization. Immunofluorescence analyses revealed an increase in NR4A2 nuclear translocation at 24 h after Cavβ4 transfection (Figure 2e,f).

### 3.3 The Cavβ4 subunit increases FOXO1 protein abundance

**FIGURE 3:**
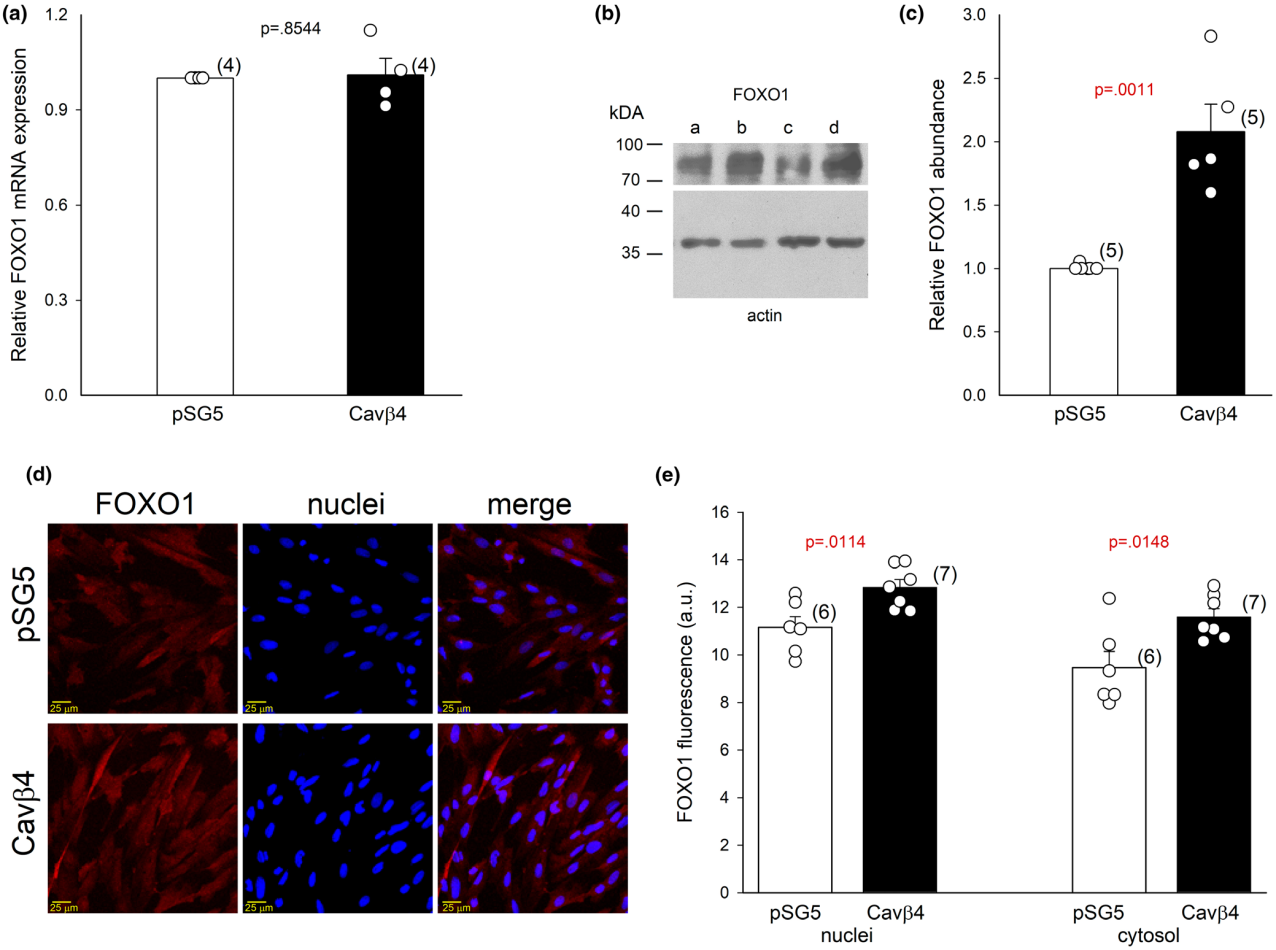
The Cavβ4 subunit increases FOXO1 protein abundance. (a) Mean (± standard error of the mean) relative FOXO1 mRNA expression under control (1.0005 ± 2.0560e^−6^) and Cavβ4 overexpression conditions (1.0105 ± 0.0520). Each symbol represents a separate experiment. (b) Representative blots of FOXO1 and GAPDH under control conditions (lanes a and c) and with Cavβ4 overexpression (lanes b and d) from two separate experiments. (c) Mean (± standard error of the mean) relative FOXO1 protein abundance under control (1.0010 ± 0.0290) and Cavβ4 overexpression conditions (2.0765 ± 0.2176). Each symbol represents a separate experiment. (d) Representative images of the immunofluorescence of H9c2 cardiomyocytes transfected with empty vector or the Cavβ4 subunit. Images show the localization of FOXO1 (red) and Hoechst‐stained nuclei (blue). (e) Mean (± standard error of the mean) nuclear FOXO1 fluorescence under control (11.1533 ± 0.4552) and Cavβ4 overexpression conditions (12.8343 ± 0.3342); FOXO1 cytosolic fluorescence under control (9.4600 ± 0.6882) and Cavβ4 overexpression conditions (11.5914 ± 0.3508). Each symbol represents a separate field, and the numbers of fields are indicated in parentheses. Calibration bar, 25 μm.

We tested the possibility that the decrease in miR‐183‐5p expression by Cavβ4 brings about an increase in FOXO1 protein abundance since it is a validated target of miR‐183‐5p. FOXO1 mRNA expression remained unchanged, whereas FOXO1 protein abundance increased significantly with Cavβ4 overexpression (Figure 3a–c). GAPDH bands in western blots remained unchanged and were used for normalization. In agreement with the western blot results, confocal analysis showed significantly increased FOXO1 nuclear and cytosolic fluorescence in Cavβ4‐transfected H9c2 cardiomyocytes (Figure 3d,e).

### 3.4 Cardiac hypertrophy regulates Cavβ4 expression

**FIGURE 4:**
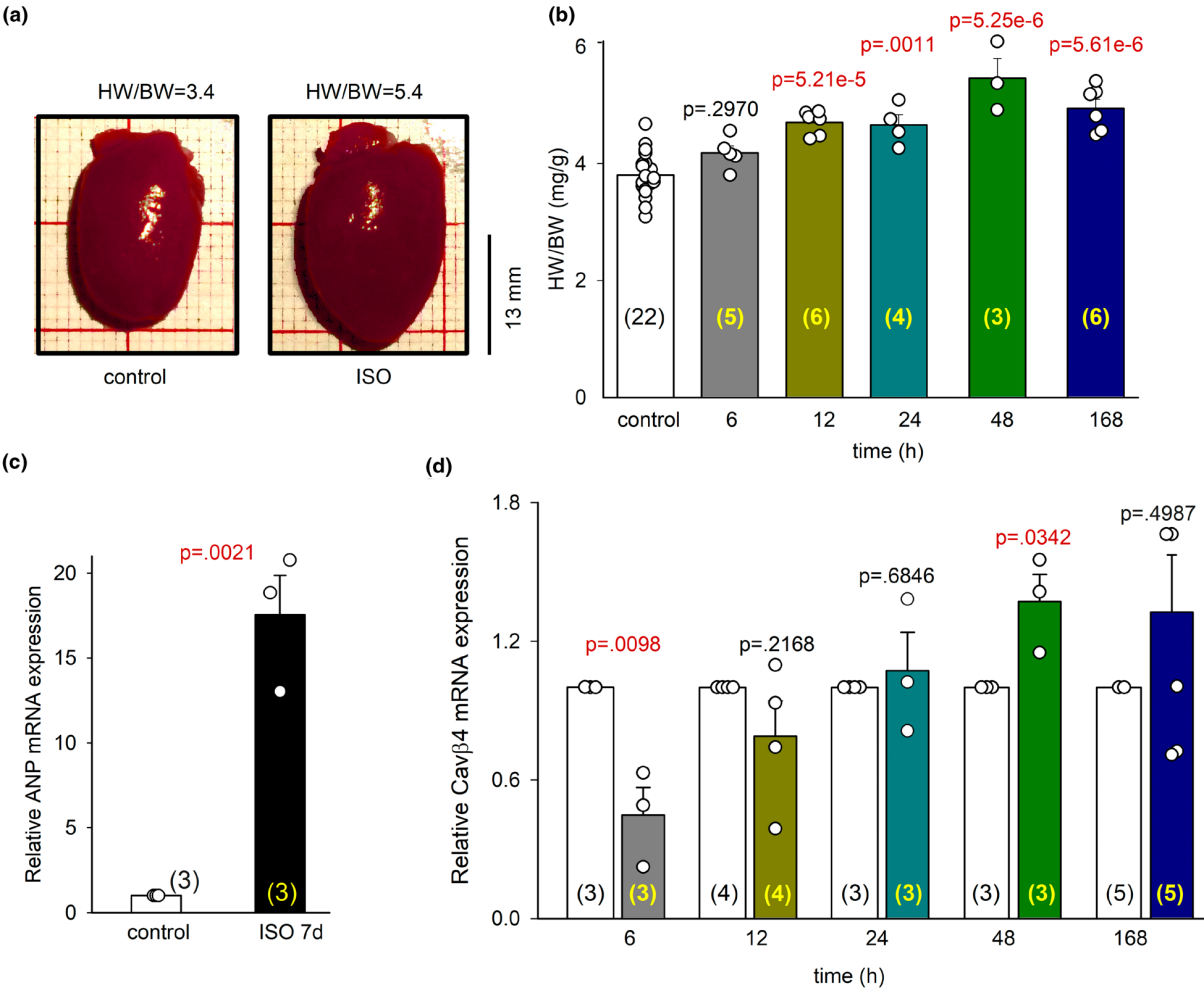
Hypertrophy regulates the expression of the Cavβ4 subunit in rats. (a) Morphology of rat hearts under control conditions (left) and after 7 days of ISO treatment (right). (b) Mean (± standard error of the mean) heart weight/body weight (HW/BW) ratios at the indicated times under control (3.8042 ± 0.0803) and ISO treatments (6 h, 4.1856 ± 0.1220; 12 h, 4.7055 ± 0.0824; 24 h, 4.6663 ± 0.1742; 48 h, 5.4640 ± 0.3408, and 168 h, 4.9498 ± 0.1553). (c) Relative ANP mRNA expression under control conditions and after 7 days of ISO treatment. (d) Relative Cavβ4 mRNA expression under control (6 h, 1.0009 ± 3.5609e‐4; 12 h, 1.0003 ± 1.5414e^−4^; 24 h, 1.0002 ± 1.7660e^−4^; 48 h, 1.0017 ± 1.6667e^−3^, and 168 h, 1.0004 ± 1.0680e^−4^) and ISO treatments (6 h, 0.4476 ± 0.1195; 12 h, 0.7898 ± 0.1525; 24 h, 1.0729 ± 0.1665; 48 h, 1.3722 ± 0.1178, and 168 h, 1.3265 ± 0.2470). Each symbol represents a separate experiment. The numbers of experiments are indicated in parentheses.

We tested the hypothesis that a decrease in the expression of the cardiac Cavβ4 subunit mRNA is produced when rats are treated with ISO. Hearts were significantly enlarged after 12 h of ISO treatment (Figure 4a). This treatment gradually increased the heart/body weight ratio, which reached a plateau after 7 days (Figure 4b). As expected, ANP expression was greatly increased after ISO treatment (Figure 4c). Cardiac Cavβ4 mRNA expression was decreased after 6 h of ISO treatment, but this effect gradually subsided and expression was increased at 48 h (Figure 4d).

## 4 DISCUSSION

Our experiments demonstrated that the Cavβ4 subunit of Cav1.2 channels antagonizes ISO‐induced hypertrophy and downregulates the expression of miR‐183‐5p in H9c2 cardiomyocytes. The Cavβ4 subunit also increased the protein expression of the antihypertrophic transcription factors FOXO1 and NR4A2. In addition, cardiac Cavβ4 mRNA expression in rats initially decreased in response to ISO treatment. The inhibition of antihypertrophic factors during pathological hypertrophy is a well‐known phenomenon. For example, glycogen synthase kinase‐3β actively inhibits hypertrophy but is phosphorylated and inhibited, allowing unchecked growth signaling in response to hypertrophic signals in cultured cardiomyocytes and in vivo in the hearts of rats subjected to pressure overload (Kerkelä et al., 2007). The regulation of Cavβ4 expression by ISO is a complex biphasic phenomenon. The transient decrease in Cavβ4 mRNA expression in rats was followed by an increase after 2 days of ISO treatment in this study, possibly reflecting a compensatory mechanism.

Among Cavβ subunits, Cavβ2 has been shown to also have antihypertrophic activity in neonatal rat cardiomyocytes (Pickel et al., 2021). However, Cavβ2 antagonizes hypertrophy by regulating the expression of the Ca^2+^‐dependent protease calpain, with no apparent miR involvement (Pickel et al., 2021). Thus, the repression of miR‐183‐5p by the Cavβ4 subunit observed in this study is a novel function of this Ca^2+^ channel subunit. The localization of the Cavβ4 subunit in the nuclei of H9c2 cardiomyocytes (Tammineni et al., 2018) and neurons (Tadmouri et al., 2012) suggests its involvement in gene expression. The subunit's repression of the tyrosine hydroxylase gene (Tadmouri et al., 2012), interferon‐β–related genes (Tammineni et al., 2018), and the CACNA1C subunit gene (Carrillo et al., 2024) has been described, but no previous report describes its repression of miRs.

Whereas an increase in FOXO1 gene expression would be accompanied by higher levels of FOXO1 mRNA, our experiments showed no change in FOXO1 mRNA expression. This finding suggests that the increase in FOXO1 protein abundance produced by Cavβ4 overexpression results from the posttranscriptional, rather than direct, regulation of FOXO1 gene expression by this subunit. The upregulation of FOXO1 at the protein level is likely related to the downregulation of miR‐183‐5p by Cavβ4. Luciferase assays and observations relating miR‐183‐5p mimics to decreases in FOXO1 protein levels have validated miR‐183‐5p's targeting of FOXO1 in H9c2 cardiomyocytes (Geng et al., 2023). miR‐183 has also been reported to repress FOXO1 expression in other systems (He et al., 2025; Ichiyama et al., 2016; Suzuki et al., 2018). The existence of more than one target for a single miR is common (Naeli et al., 2023). In this regard, the observation of increased luciferase activity in HEK‐293 cells treated with an miR‐183 inhibitor (Gong et al., 2021) suggests that miR‐183‐5p also binds and regulates NR4A2 mRNA. Given our observation of miR‐183‐5p down‐regulation by Cavβ4 expression and the previous validation of FOXO1 and NR4A2 as targets of this miR, we propose that the downregulation of miR‐183‐5p plays a role in the increase in FOXO1 and NR4A2 protein abundance with Cavβ4 overexpression.

The hallmark of cardiac hypertrophy is an increase in the size of cardiomyocytes. Two types of hypertrophies have been described, physiological and pathological. Both are regulated by distinct signaling pathways (Martin et al., 2023). The role of FOXO1 in both types of cardiac hypertrophy is complex. The expression of FOXO1 does not change after exercise‐induced cardiac hypertrophy, but a cardiac specific knock out of FOXO1 prevents physiological hypertrophy in mice (Weeks et al., 2021). On the other hand, in a mouse model of pathological hypertrophy induced by chronic application of isoproterenol, expression of an adenovirus construct that expresses constitutively active FOXO1 completely prevents the development of cardiac hypertrophy (Martin et al., 2025).

Several pieces of evidence support the conclusion that the increase in FOXO1 protein abundance is involved in the antihypertrophic effect of the Cavβ4 subunit in our experiments. First, FOXO1 activation is sufficient to prevent pathological hypertrophy induced by an adrenergic agonist (Martin et al., 2025). Second, the activity of FOXO1 and FOXO3 protects the heart from pathological hypertrophy by preventing an increase in cardiomyocyte size under stress conditions (Orea‐Soufi et al., 2022; Sengupta et al., 2009). Third, multiple hypertrophic ligands, including ISO, inhibit the expression of FOXO transcription factors in cardiomyocytes (Ni et al., 2006). Furthermore, the FOXO target gene product atrogin‐1 ubiquitinates and degrades calcineurin, preventing Akt‐mediated hypertrophic signaling (Ronnebaum & Patterson, 2010).

The increase in NR4A2 protein abundance that we observed after Cavβ4 overexpression is also likely involved in the antihypertrophic action of this subunit. NR4A2 acts as a negative regulator of the β‐adrenergic receptor–mediated growth response, preventing the hypertrophic growth of adult cardiomyocytes in response to ISO (Ashraf et al., 2019), and it also protects cardiomyocytes against myocardial infarction injury (Liu et al., 2018). However, the actions of NR4A2 are also complex since extremely high and chronic overexpression of NR4A2 in mice leads to maladaptive changes resulting in cardiac hypertrophy and left ventricular dilation (Ashraf et al., 2022). Taken together, our experimental data and previous work on the roles of FOXO1 and NR4A2 in cardiac hypertrophy at the cellular level suggest that the antihypertrophic activity of the Cavβ4 subunit in H9c2 cardiomyocytes observed in this study is related to the observed decrease in the miR‐183‐5p level after Cavβ4 overexpression.

Although the mechanism underlying the repression of miR‐183‐5p by Cavβ4 remains to be elucidated, cAMP responsive element binding protein 1 (CREB1) may be involved. CREB1 targets miR‐183‐5p, as evidenced by its direct binding to the miR‐183‐5p promoter region in HEK‐293 cells in chromatin immunoprecipitation assays, which results in the transcriptional inhibition of its expression (Jin et al., 2025). The phosphorylation of CREB1 is a central regulatory mechanism that increases its transcriptional activity (Mayr & Montminy, 2001), and its dephosphorylation depends on protein phosphatase 2A (PP2A) and is critically dependent on B56δ, a regulatory subunit of PP2A (Zhang et al., 2024). Direct interaction between Cavβ4 and B56δ has been demonstrated by coimmunoprecipitation experiments (Tadmouri et al., 2012). This interaction's functional significance is presently unknown, but it is possible that Cavβ4, when interacting with PP2A, might reduce its activity, increasing CREB1 phosphorylation and transcriptional activity, thereby reducing the miR‐183‐5p level. Another possibility would involve ZNF304, a transcription factor that has been experimentally validated as a transcriptional repressor of miR‐183 in other systems (Ren et al., 2021). However, its expression in heart muscle is low (https://www.proteinatlas.org/ENSG00000131845‐ZNF304/tissue) and no protein–protein interactions of this factor or associated proteins with Cavβ4 have been described. Therefore, its role in miR‐183‐5p regulation in our experiments is uncertain. Further experimentation is required to characterize the exact mechanism of miR‐183‐5p repression by the Cavβ4 subunit.

Although our experiments suggest that miR‐183‐5p, FOXO1, and NR4A2 play roles in the antihypertrophic effects of Cavβ4 in vitro, several limitations must be taken into account. First, whether Cavβ4 has similar antihypertrophic actions in vivo needs to be assessed in gain‐ and loss‐of‐function studies and with the examination of cardiac‐specific changes in Cavβ4 protein abundance in transgenic animals. Second, we examined a single miR species in this study, and the contributing roles of other miRs cannot be ruled out. For example, FOXO1 is also a target of miR‐144, miR‐96, and miR‐21 (Fendler et al., 2013; Lin et al., 2020; Song et al., 2015) and NR4A2 is a target of miR‐34, miR‐132, and miR‐212 (Beard et al., 2016; Liu et al., 2018; Yang et al., 2012). Additional experimentation using microarrays or deep RNA sequencing methods could reveal a more complex picture of the antihypertrophic regulatory mechanisms of Cavβ4.

In conclusion, our experiments demonstrate that the Ca^2+^ channel subunit Cavβ4 acts as an antihypertrophic factor in cardiac cells in vitro and decreases the expression of miR‐183‐5p while increasing the transcription factors FOXO1 and NR4A2 protein abundance. These data are consistent with Cavβ4's triggering of an antihypertrophic response to stress stimuli, in which miR‐183‐5p, FOXO1, and NR4A2 play roles. Given the clinical importance of protecting the heart from pathological hypertrophy, a detailed understanding of the cellular mechanisms underlying the mitigation of hypertrophy by endogenous phenomena is relevant to the development of cardioprotective therapies.

## AUTHOR CONTRIBUTIONS

**Elba D. Carrillo:** Conceptualization; data curation; formal analysis; investigation; methodology; validation. **Erick Galicia:** Data curation; formal analysis; investigation; methodology. **María C. García:** Conceptualization; data curation; formal analysis; methodology; validation. **Jorge A. Sánchez:** Conceptualization; data curation; formal analysis; funding acquisition; investigation; project administration; resources; software; supervision; validation; visualization.

## FUNDING INFORMATION

This work was supported in part by the Ministry of Science, Humanities, Technology and Innovation of Mexico (CBF‐2025‐I‐125) to J.A.S, E.G. was supported by a fellowship from Mexico's National Council of Humanities, Sciences, and Technologies.

## CONFLICT OF INTEREST STATEMENT

The authors declare that they have no known competing financial interest or personal relationship that could have appeared to influence the work reported in this paper.

## ETHICS STATEMENT

This study was conducted in accordance with institutional, national, and ethical guidelines for the care and use of laboratory animals. All animal procedures were reviewed and approved by the Institutional Animal Care and Use Committee at Center for Research and Advanced Studies of the National Polytechnic Institute, Mexico. The study was designed to minimize animal distress, ensure appropriate housing and monitoring, and use scientifically justified sample sizes for histological and cellular analyses. No human participants or human‐derived materials were used in this study.

## CONSENT

All the authors approved the final version of the manuscript and consented to its publication.

## Supporting information


**Table S1.** Antibodies used for Western blots and Immunocytochemistry.

## Data Availability

The study data are available from the corresponding author, Jorge A. Sánchez, upon reasonable request.

## References

[phy271037-bib-0001] Ashraf, S. , Hegazy, Y. K. , & Harmancey, R. (2019). Nuclear receptor subfamily 4 group a member 2 inhibits activation of ERK signaling and cell growth in response to β‐adrenergic stimulation in adult rat cardiomyocytes. American Journal of Physiology‐Cell Physiology, 317, C513–C524. 10.1152/ajpcell.00526.2018 31188636 PMC6766613

[phy271037-bib-0002] Ashraf, S. , Taegtmeyer, H. , & Harmancey, R. (2022). Prolonged cardiac NR4A2 activation causes dilated cardiomyopathy in mice. Basic Research in Cardiology, 117, 33. 10.1007/s00395-022-00942-7 35776225 PMC9249728

[phy271037-bib-0003] Baviskar, S. N. (2011). A Quick & Automated Method for measuring cell area using ImageJ. The American Biology Teacher, 73, 554–556. 10.1525/abt.2011.73.9.9

[phy271037-bib-0004] Beard, J. A. , Tenga, A. , Hills, J. , Hoyer, J. D. , Cherian, M. T. , Wang, Y.‐D. , & Chen, T. (2016). The orphan nuclear receptor NR4A2 is part of a p53–microRNA‐34 network. Scientific Reports, 6, 25108. 10.1038/srep25108 27121375 PMC4848494

[phy271037-bib-0005] Bers, D. M. (2002). Cardiac excitation‐contraction coupling. Nature, 415, 198–205. 10.1038/415198a 11805843

[phy271037-bib-0006] Bradford, M. M. (1976). A rapid and sensitive method for the quantitation of microgram quantities of protein utilizing the principle of protein‐dye binding. Analytical Biochemistry, 72, 248–254. 10.1016/0003-2697(76)90527-3 942051

[phy271037-bib-0007] Carrillo, E. D. , Alvarado, J. A. , Hernández, A. , Lezama, I. , García, M. C. , & Sánchez, J. A. (2024). Thyroid hormone upregulates Cav1.2 channels in cardiac cells via the downregulation of the channels' β4 subunit. International Journal of Molecular Sciences, 25, 10798. 10.3390/ijms251910798 39409130 PMC11476369

[phy271037-bib-0008] Castellano, A. , Wei, X. , Birnbaumer, L. , & Perez‐Reyes, E. (1993). Cloning and expression of a neuronal calcium channel beta subunit. The Journal of Biological Chemistry, 268, 12359–12366. 7685340

[phy271037-bib-0009] Dolphin, A. C. (2016). Voltage‐gated calcium channels and their auxiliary subunits: Physiology and pathophysiology and pharmacology. The Journal of Physiology, 594, 5369–5390. 10.1113/JP272262 27273705 PMC5043047

[phy271037-bib-0010] Fendler, A. , Jung, M. , Stephan, C. , Erbersdobler, A. , Jung, K. , & Yousef, G. M. (2013). The antiapoptotic function of miR‐96 in prostate cancer by inhibition of FOXO1. PLoS One, 8, e80807. 10.1371/journal.pone.0080807 24260486 PMC3834337

[phy271037-bib-0011] Geng, T. , Xu, Z. , Xing, J. , Yuan, Y. , & Liu, J. (2023). Knockdown of lncRNA SNHG16 attenuates myocardial ischemia‐reoxygenation injury via targeting miR‐183/FOXO1 axis. Experimental and Therapeutic Medicine, 25, 106. 10.3892/etm.2023.11805 36778043 PMC9909512

[phy271037-bib-0012] Gong, F.‐H. , Long, L. , Yang, Y.‐S. , Shen, D.‐H. , Zhang, Y.‐S. , Wang, X.‐S. , Zhang, X. P. , & Xiao, X. Q. (2021). Attenuated macrophage activation mediated by microRNA‐183 knockdown through targeting NR4A2. Experimental and Therapeutic Medicine, 21, 300. 10.3892/etm.2021.9731 33717243 PMC7885059

[phy271037-bib-0013] Han, Y. , Nie, J. , Wang, D. W. , & Ni, L. (2022). Mechanism of histone deacetylases in cardiac hypertrophy and its therapeutic inhibitors. Frontiers in Cardiovascular Medicine, 9, 931475. 10.3389/fcvm.2022.931475 35958418 PMC9360326

[phy271037-bib-0014] He, T. , Xue, X. , & Shi, J. (2025). Downregulation of FOXO1 in PCOS granulosa cells: A direct target of microRNA‐183. Reproductive Sciences, 32, 2467–2473. 10.1007/s43032-025-01886-8 40447928

[phy271037-bib-0015] Ichiyama, K. , Gonzalez‐Martin, A. , Kim, B.‐S. , Jin, H. Y. , Jin, W. , Xu, W. , Sabouri‐Ghomi, M. , Xu, S. , Zheng, P. , Xiao, C. , & Dong, C. (2016). The MicroRNA‐183‐96‐182 cluster promotes T helper 17 cell pathogenicity by negatively regulating transcription factor Foxo1 expression. Immunity, 44, 1284–1298. 10.1016/j.immuni.2016.05.015 27332731 PMC4918454

[phy271037-bib-0016] Jin, F. , Long, K. , & Zhang, J. (2025). Transcription factor CREB1 promotes malignant growth of osteosarcoma cells by inhibiting miRNA‐183‐5p and regulating PROX1 expression. Molecular Biology Reports, 52, 647. 10.1007/s11033-025-10741-7 40579642

[phy271037-bib-0017] Kelley, J. B. , & Paschal, B. M. (2019). Fluorescence‐based quantification of nucleocytoplasmic transport. Methods, 157, 106–114. 10.1016/j.ymeth.2018.11.002 30419335 PMC7041306

[phy271037-bib-0018] Kerkelä, R. , Woulfe, K. , & Force, T. (2007). Glycogen synthase kinase‐3beta—actively inhibiting hypertrophy. Trends in Cardiovascular Medicine, 17, 91–96. 10.1016/j.tcm.2007.01.004 17418370

[phy271037-bib-0019] Lin, W. , Tang, Y. , Zhao, Y. , Zhao, J. , Zhang, L. , Wei, W. , & Chen, J. (2020). MiR‐144‐3p targets FoxO1 to reduce its regulation of adiponectin and promote adipogenesis. Frontiers in Genetics, 11, 603144. 10.3389/fgene.2020.603144 33381152 PMC7767994

[phy271037-bib-0020] Liu, H. , Liu, P. , Shi, X. , Yin, D. , & Zhao, J. (2018). NR4A2 protects cardiomyocytes against myocardial infarction injury by promoting autophagy. Cell Death Discovery, 4, 27. 10.1038/s41420-017-0011-8 PMC5841341 29531824

[phy271037-bib-0021] Livak, K. J. , & Schmittgen, T. D. (2001). Analysis of relative gene expression data using real‐time quantitative PCR and the 2(‐Delta Delta C(T)) method. Methods, 25, 402–408. 10.1006/meth.2001.1262 11846609

[phy271037-bib-0022] Makarewich, C. A. , Correll, R. N. , Gao, H. , Zhang, H. , Yang, B. , Berretta, R. M. , Rizzo, V. , Molkentin, J. D. , & Houser, S. R. (2012). A caveolae‐targeted L‐type Ca^2^+ channel antagonist inhibits hypertrophic signaling without reducing cardiac contractility. Circulation Research, 110, 669–674. 10.1161/CIRCRESAHA.111.264028 22302787 PMC3324037

[phy271037-bib-0023] Martin, T. G. , Juarros, M. A. , & Leinwand, L. A. (2023). Regression of cardiac hypertrophy in health and disease: Mechanisms and therapeutic potential. Nature Reviews. Cardiology, 20, 347–363. 10.1038/s41569-022-00806-6 36596855 PMC10121965

[phy271037-bib-0024] Martin, T. G. , Langer, S. J. , Crocini, C. , Chung, E. , & Leinwand, L. A. (2025). Activation of FoxO1 prevents and reverses cardiac hypertrophy from diverse stimuli. Journal of Molecular and Cellular Cardiology, 205, 62–67. 10.1016/j.yjmcc.2025.06.008 40553761 PMC12969049

[phy271037-bib-0025] Mayr, B. , & Montminy, M. (2001). Transcriptional regulation by the phosphorylation‐dependent factor CREB. Nature Reviews. Molecular Cell Biology, 2, 599–609. 10.1038/35085068 11483993

[phy271037-bib-0026] McLoughlin, H. S. , Wan, J. , Spengler, R. M. , Xing, Y. , & Davidson, B. L. (2014). Human‐specific microRNA regulation of FOXO1: Implications for microRNA recognition element evolution. Human Molecular Genetics, 23, 2593–2603. 10.1093/hmg/ddt655 24368418

[phy271037-bib-0027] Morisco, C. , Zebrowski, D. C. , Vatner, D. E. , Vatner, S. F. , & Sadoshima, J. (2001). Beta‐adrenergic cardiac hypertrophy is mediated primarily by the beta(1)‐subtype in the rat heart. Journal of Molecular and Cellular Cardiology, 33, 561–573. 10.1006/jmcc.2000.1332 11181023

[phy271037-bib-0028] Mukherjee, R. , & Spinale, F. G. (1998). L‐type calcium channel abundance and function with cardiac hypertrophy and failure: A review. Journal of Molecular and Cellular Cardiology, 30, 1899–1916. 10.1006/jmcc.1998.0755 9799645

[phy271037-bib-0029] Myers, S. A. , Eriksson, N. , Burow, R. , Wang, S.‐C. M. , & Muscat, G. E. O. (2009). Beta‐adrenergic signaling regulates NR4A nuclear receptor and metabolic gene expression in multiple tissues. Molecular and Cellular Endocrinology, 309, 101–108. 10.1016/j.mce.2009.05.006 19465082

[phy271037-bib-0030] Naeli, P. , Winter, T. , Hackett, A. P. , Alboushi, L. , & Jafarnejad, S. M. (2023). The intricate balance between microRNA‐induced mRNA decay and translational repression. The FEBS Journal, 290, 2508–2524. 10.1111/febs.16422 35247033

[phy271037-bib-0031] Ni, Y. G. , Berenji, K. , Wang, N. , Oh, M. , Sachan, N. , Dey, A. , Cheng, J. , Lu, G. , Morris, D. J. , Castrillon, D. H. , Gerard, R. D. , Rothermel, B. A. , & Hill, J. A. (2006). Foxo transcription factors blunt cardiac hypertrophy by inhibiting calcineurin signaling. Circulation, 114, 1159–1168. 10.1161/CIRCULATIONAHA.106.637124 16952979 PMC4118290

[phy271037-bib-0032] Nishida, M. , Mi, X. , Ishii, Y. , Kato, Y. , & Nishimura, A. (2024). Cardiac remodeling: Novel pathophysiological mechanisms and therapeutic strategies. Journal of Biochemistry, 176, 255–262. 10.1093/jb/mvae031 38507681

[phy271037-bib-0033] Orea‐Soufi, A. , Paik, J. , Bragança, J. , Donlon, T. A. , Willcox, B. J. , & Link, W. (2022). FOXO transcription factors as therapeutic targets in human diseases. Trends in Pharmacological Sciences, 43, 1070–1084. 10.1016/j.tips.2022.09.010 36280450 PMC12194985

[phy271037-bib-0034] Oussaief, L. , Fendri, A. , Chane‐Woon‐Ming, B. , Poirey, R. , Delecluse, H.‐J. , Joab, I. , & Pfeffer, S. (2015). Modulation of MicroRNA cluster miR‐183‐96‐182 expression by Epstein‐Barr virus latent membrane protein 1. Journal of Virology, 89, 12178–12188. 10.1128/JVI.01757-15 26401047 PMC4645329

[phy271037-bib-0035] Pickel, S. , Cruz‐Garcia, Y. , Bandleon, S. , Barkovits, K. , Heindl, C. , Völker, K. , Abeßer, M. , Pfeiffer, K. , Schaaf, A. , Marcus, K. , Eder‐Negrin, P. , Kuhn, M. , & Miranda‐Laferte, E. (2021). The β2‐subunit of voltage‐gated calcium channels regulates cardiomyocyte hypertrophy. Frontiers in Cardiovascular Medicine, 8, 704657. 10.3389/fcvm.2021.704657 34307509 PMC8292724

[phy271037-bib-0036] Ren, L.‐X. , Zeng, B.‐W. , Zhu, M. , Zhao, A.‐N. , Shi, B. , Zhang, H. , Wang, D. D. , Gu, J. F. , & Yang, Z. (2021). A novel ZNF304/miR‐183‐5p/FOXO4 pathway regulates cell proliferation in clear cell renal carcinoma. Frontiers in Oncology, 11, 710525. 10.3389/fonc.2021.710525 34692488 PMC8529286

[phy271037-bib-0037] Ronnebaum, S. M. , & Patterson, C. (2010). The FoxO family in cardiac function and dysfunction. Annual Review of Physiology, 72, 81–94. 10.1146/annurev-physiol-021909-135931 PMC2908381 20148668

[phy271037-bib-0038] Saliminejad, K. , Khorram Khorshid, H. R. , Soleymani Fard, S. , & Ghaffari, S. H. (2019). An overview of microRNAs: Biology, functions, therapeutics, and analysis methods. Journal of Cellular Physiology, 234, 5451–5465. 10.1002/jcp.27486 30471116

[phy271037-bib-0039] Sengupta, A. , Molkentin, J. D. , & Yutzey, K. E. (2009). FoxO transcription factors promote autophagy in cardiomyocytes. The Journal of Biological Chemistry, 284, 28319–28331. 10.1074/jbc.M109.024406 19696026 PMC2788882

[phy271037-bib-0040] Song, W. , Li, Q. , Wang, L. , & Wang, L. (2015). Modulation of FoxO1 expression by miR‐21 to promote growth of pancreatic ductal adenocarcinoma. Cellular Physiology and Biochemistry, 35, 184–190. 10.1159/000369686 25591761

[phy271037-bib-0041] Stark, T. J. , Arnold, J. D. , Spector, D. H. , & Yeo, G. W. (2012). High‐resolution profiling and analysis of viral and host small RNAs during human cytomegalovirus infection. Journal of Virology, 86, 226–235. 10.1128/JVI.05903-11 22013051 PMC3255895

[phy271037-bib-0042] Suzuki, R. , Amatya, V. J. , Kushitani, K. , Kai, Y. , Kambara, T. , & Takeshima, Y. (2018). miR‐182 and miR‐183 promote cell proliferation and invasion by targeting FOXO1 in mesothelioma. Frontiers in Oncology, 8, 446. 10.3389/fonc.2018.00446 30406026 PMC6204457

[phy271037-bib-0043] Tadmouri, A. , Kiyonaka, S. , Barbado, M. , Rousset, M. , Fablet, K. , Sawamura, S. , Bahembera, E. , Pernet‐Gallay, K. , Arnoult, C. , Miki, T. , Sadoul, K. , Gory‐Faure, S. , Lambrecht, C. , Lesage, F. , Akiyama, S. , Khochbin, S. , Baulande, S. , Janssens, V. , Andrieux, A. , … de Waard, M. (2012). Cacnb4 directly couples electrical activity to gene expression, a process defective in juvenile epilepsy. The EMBO Journal, 31, 3730–3744. 10.1038/emboj.2012.226 22892567 PMC3442274

[phy271037-bib-0044] Tammineni, E. R. , Carrillo, E. D. , Soto‐Acosta, R. , Angel‐Ambrocio, A. H. , García, M. C. , Bautista‐Carbajal, P. , Angel, R. M. , & Sánchez, J. A. (2018). The β4 subunit of Cav1.2 channels is required for an optimal interferon response in cardiac muscle cells. Science Signaling, 11, eaaj1676. 10.1126/scisignal.aaj1676 30538175

[phy271037-bib-0045] Urbánek, P. , & Klotz, L.‐O. (2017). Posttranscriptional regulation of FOXO expression: microRNAs and beyond. British Journal of Pharmacology, 174, 1514–1532. 10.1111/bph.13471 26920226 PMC5446586

[phy271037-bib-0046] Velusamy, P. , Mohan, T. , Ravi, D. B. , Kishore Kumar, S. N. , Srinivasan, A. , Chakrapani, L. N. , Singh, A. , Varadharaj, S. , & Kalaiselvi, P. (2020). Targeting the Nrf2/ARE Signalling pathway to mitigate isoproterenol‐induced cardiac hypertrophy: Plausible role of Hesperetin in redox homeostasis. Oxidative Medicine and Cellular Longevity, 2020, 9568278. 10.1155/2020/9568278 32952852 PMC7482027

[phy271037-bib-0047] Warkman, A. S. , Whitman, S. A. , Miller, M. K. , Garriock, R. J. , Schwach, C. M. , Gregorio, C. C. , & Krieg, P. A. (2012). Developmental expression and cardiac transcriptional regulation of Myh7b, a third myosin heavy chain in the vertebrate heart. Cytoskeleton (Hoboken), 69, 324–335. 10.1002/cm.21029 22422726 PMC4734749

[phy271037-bib-0048] Weeks, K. L. , Tham, Y. K. , Yildiz, S. G. , Alexander, Y. , Donner, D. G. , Kiriazis, H. , Harmawan, C. A. , Hsu, A. , Bernardo, B. C. , Matsumoto, A. , DePinho, R. A. , Abel, E. D. , Woodcock, E. A. , & McMullen, J. R. (2021). FoxO1 is required for physiological cardiac hypertrophy induced by exercise but not by constitutively active PI3K. American Journal of Physiology. Heart and Circulatory Physiology, 320, H1470–H1485. 10.1152/ajpheart.00838.2020 33577435

[phy271037-bib-0049] Yang, D. , Li, T. , Wang, Y. , Tang, Y. , Cui, H. , Tang, Y. , Zhang, X. , Chen, D. , Shen, N. , & le, W. (2012). miR‐132 regulates the differentiation of dopamine neurons by directly targeting Nurr1 expression. Journal of Cell Science, 125, 1673–1682. 10.1242/jcs.086421 22328530

[phy271037-bib-0050] Yu, W. , Chen, C. , & Cheng, J. (2020). The role and molecular mechanism of FoxO1 in mediating cardiac hypertrophy. ESC Heart Failure, 7, 3497–3504. 10.1002/ehf2.13065 33089967 PMC7755013

[phy271037-bib-0051] Zhang, W. , Yano, N. , Deng, M. , Mao, Q. , Shaw, S. K. , & Tseng, Y.‐T. (2011). β‐Adrenergic receptor‐PI3K signaling crosstalk in mouse heart: Elucidation of immediate downstream signaling cascades. PLoS One, 6, e26581. 10.1371/journal.pone.0026581 22028912 PMC3197531

[phy271037-bib-0052] Zhang, Y. , Jiang, H. , Yin, H. , Zhao, X. , & Zhang, Y. (2024). Emerging roles of B56 phosphorylation and binding motif in PP2A‐B56 holoenzyme biological function. International Journal of Molecular Sciences, 25, 3185. 10.3390/ijms25063185 38542160 PMC10970375

