# Supplementary material for: The Cavβ4 subunit of Cav1.2 channels antagonizes isoproterenol‐induced hypertrophy in rat cardiac muscle cells by down‐regulating miR‐183‐5p
Source: Physiol Rep. 2026 Jul 28;14(15):e71037. doi: 10.14814/phy2.71037 (PMC13415754; doi:10.14814/phy2.71037)
Supplement: Supplementary file 1 — Table S1. Antibodies used for Western blots and Immunocytochemistry. [file PHY2-14-e71037-s001.docx]

**The Cavβ4 subunit of Cav1.2 channels antagonizes isoproterenol-induced hypertrophy in rat cardiac muscle cells by down-regulating miR-183-5p**

Elba D. Carrillo, Erick Galicia, María C. García, and Jorge A. Sánchez

Supplementary file:

Supplementary table S1: Antibodies used for Western blots and Immunocytochemistry

**Supplementary table S1: Antibodies used for Western blots and Immunocytochemistry**

| **Primary antibodies** | | | | | | | |
| --- | --- | --- | --- | --- | --- | --- | --- |
| **Target** | **Host** | **Clonality** | **Vendor** | **Catalogue #, RRID** | | **Dilution WB** | **Dilution**  **ICC** |
| Cavβ4 | Rabbit | polyclonal | AB clonal | A4304 RRID:AB_2768673 | | 1:1000 | - |
| FOXO1 | Rabbit | monoclonal | Cell Signaling | C29H4 RRID:AB_2106495 | | 1:500 | 1:200 |
| FOXO1 | Mouse | monoclonal | Gene Tex | GTX60558 RRID:AB_3750393 | | 1:250 | - |
| NR4A2 | Rabbit | polyclonal | Pro Intech | 10-975-2-AB RRID:AB_2153760 | | 1:1000 | 1:500 |
| GAPDH | Mouse | monoclonal | Sigma-Aldrich | G8795 RRID:AB_1078991 | | 1:2500 | - |
| β-Actin | Mouse | monoclonal | Santa Cruz | SC-47778 RRID:AB_626632 | | 1:500 | - |
| **Secondary antibodies** | | | | | | | |
| **Target** | **Host** | **Clonality** | **Vendor** | **Catalogue #, RRID** | **Dilution WB** | | **Dilution**  **ICC** |
| HRP-conjugate- Goat anti-Mouse IgG (H+L) | Mouse | polyclonal | Thermo-Fisher | 81-6520 | 1:800,000 | | - |
| HRP-conjugate-Goat Anti-Rabbit IgG (H+L) | Rabbit | polyclonal | Thermo-Fisher | 81-6120 RRID:AB_87750 | 1:800,000 | | - |
| Alexa Fluor 555-conjugate- Donkey anti-Rabbit IgG (H+L) | Rabbit | polyclonal | Thermo-Fisher | A31572 RRID:AB_162543 | - | | 1:300 |
